# Dialectical Behaviour Therapy for Eating Disorders With Comorbid Borderline Personality Disorder: A Systematic Review and Clinical Implications

**DOI:** 10.1002/cpp.70315

**Published:** 2026-08-04

**Authors:** Semina Z. Ozdemir, Ceren Turkan, Leyla S. Dikmen, Oya Mortan Sevi

**Affiliations:** ^1^ Department of Psychology Yasar University Izmir Türkiye

**Keywords:** borderline personality disorder, comorbidity, dialectical behaviour therapy, eating disorders, systematic review

## Abstract

**Objective:**

Borderline personality disorder (BPD) and eating disorders (EDs) frequently co‐occur and are associated with heightened clinical severity and emotion dysregulation. This systematic review aimed to synthesize empirical evidence on the effectiveness of dialectical behaviour therapy (DBT) in improving eating disorder outcomes among individuals with comorbid BPD and EDs.

**Method:**

A systematic literature search was conducted across Wiley, Web of Science, SpringerLink and Scopus databases in accordance with PRISMA 2020 guidelines. Studies were included if they examined DBT‐based interventions in samples with comorbid BPD and EDs and reported quantitative outcomes. Eight studies met inclusion criteria. Risk of bias was assessed using Joanna Briggs Institute (JBI) checklists, and a structured narrative synthesis was performed.

**Results:**

Across studies, DBT‐based interventions were associated with reductions in eating disorder symptom severity, maladaptive eating behaviours (e.g., binge eating and purging) and self‐harm. Baseline BPD symptom severity did not consistently predict poorer outcomes and, in some cases, was associated with greater early improvements. However, substantial heterogeneity in study design, sample characteristics and outcome measures was observed, and methodological quality varied across studies.

**Conclusions:**

DBT appears to be a clinically relevant and promising transdiagnostic intervention for individuals with comorbid BPD and EDs. Nevertheless, the current evidence base is limited by methodological constraints, highlighting the need for well‐designed randomized controlled trials and mechanism‐focused research. These findings have important implications for clinical practice, supporting the use of DBT as a transdiagnostic intervention in complex comorbid populations.

## Summary

- DBT may reduce eating disorder symptoms and self‐harm in individuals with comorbid BPD and EDs.
- Emotion regulation appears to be a central mechanism underlying treatment improvement.
- DBT may be especially useful for clinically high‐risk and treatment‐resistant populations.
- Comorbid BPD symptoms do not necessarily predict poorer ED treatment outcomes.
- Transdiagnostic DBT approaches may support long‐term clinical stabilization.

## 1 Introduction

Borderline personality disorder (BPD) and eating disorders (EDs), even when considered separately, are psychiatric disorders associated with substantial functional impairment, emotion dysregulation and serious clinical consequences. BPD is characterized by affective instability, impulsive behaviour and marked interpersonal difficulties and is associated with elevated risk of self‐harm and suicide (Leichsenring et al. 2024; Paris 2019). EDs include anorexia nervosa (AN), bulimia nervosa (BN), binge‐eating disorder (BED) and other specified feeding or eating disorder (OSFED) and are associated with considerable physical and psychological morbidity as well as elevated mortality risk (van Hoeken and Hoek 2020). Although the transition from DSM‐IV to DSM‐5 introduced BED as a distinct diagnosis and replaced the broad EDNOS category with OSFED, clinically meaningful heterogeneity in symptom presentation and severity remains across ED diagnoses (Call et al. 2013).

Comorbidity between BPD and EDs is clinically important and relatively common. A recent meta‐analysis reported that approximately 30% of individuals with BPD meet criteria for an ED, with OSFED, BED and BN being particularly prevalent (Paudex et al. 2025). Individuals with co‐occurring BPD and EDs often present with more severe psychopathology than those without this comorbidity, including greater alexithymia, anxiety and depressive symptoms and more severe disordered eating attitudes (Khosravi 2020). Shared features, including affective instability, impulsivity, identity disturbance and emotion‐driven eating, may contribute to the overlap between these conditions and complicate the clinical course (Sansone and Sansone 2011; Loxton and Gleaves 2025). Thus, BPD–ED comorbidity represents a clinically complex presentation that may require interventions capable of addressing both eating‐related symptoms and broader difficulties in emotion regulation.

Dialectical behaviour therapy (DBT) may be particularly relevant for this population because it was originally developed to treat pervasive emotion dysregulation, chronic suicidality, self‐harm and behavioural dyscontrol in individuals with borderline personality disorder (Linehan 1993, 2015). Accordingly, DBT targets emotion dysregulation, impulsive behaviour, self‐harm and interpersonal difficulties through a balance of acceptance‐ and change‐oriented strategies. Randomized controlled trials and meta‐analytic findings indicate that DBT reduces self‐harm, suicidal behaviours and other forms of self‐directed violence among individuals with BPD (DeCou et al. 2019). DBT‐based interventions have also been associated with reductions in binge‐eating frequency and overall ED psychopathology, particularly in BED and BN presentations (Safer et al. 2010; Rozakou‐Soumalia et al. 2021). DBT integrates acceptance‐ and change‐oriented strategies and includes individual therapy, group skills training, telephone coaching and therapist consultation teams (Linehan 1993, 2015). Its core skills modules, including mindfulness, distress tolerance, emotion regulation and interpersonal effectiveness, may help individuals manage affect‐driven eating behaviours, while its treatment hierarchy prioritizes life‐threatening and therapy‐interfering behaviours before quality‐of‐life targets, including ED symptoms (Leichsenring et al. 2024).

Although research on DBT for BPD and EDs has expanded, most systematic reviews and meta‐analyses have examined these conditions separately or have included comorbid BPD–ED presentations only within broader samples (DeCou et al. 2019; Paudex et al. 2025; Rozakou‐Soumalia et al. 2021). However, studies focusing specifically on individuals with both diagnoses remain limited and vary considerably in sample characteristics, treatment setting, DBT adaptations and outcome measures. This heterogeneity makes it difficult to determine the extent to which DBT is associated with ED‐related improvement in this comorbid population, whether BPD symptom severity influences treatment response and which treatment components may be most relevant.

The present systematic review synthesizes the available empirical evidence on DBT and DBT‐based interventions for individuals with comorbid BPD and EDs. It examines reported ED‐related outcomes, BPD‐related and self‐harm outcomes and methodological features of the included studies. To our knowledge, this is the first systematic review to focus exclusively on DBT outcomes in individuals with comorbid BPD and EDs. Accordingly, this review addresses the following research question: Does dialectical behaviour therapy improve eating disorder–related outcomes in individuals with comorbid borderline personality disorder?

## 2 Method

A systematic literature search was conducted in the Wiley, Web of Science, SpringerLink and Scopus databases to identify peer‐reviewed articles published in English or Turkish. The review was conducted in accordance with the PRISMA 2020 guidelines for systematic reviews.

For Web of Science and Scopus, two complementary search strings were applied. These yielded 588 and 128 records from Web of Science and 975 and 68 records from Scopus, respectively (total: 716 and 1043 records).

The search strategy included the following keywords and their combinations: “dialectical behavior therapy AND borderline personality disorder AND eating disorders”; “dialectical behavior therapy AND binge eating disorder OR bulimia nervosa AND borderline personality disorder”; and “dialectical behavior therapy OR DBT AND borderline personality disorder OR BPD AND eating disorders OR ED OR binge eating disorder OR bulimia.” These search strings were adapted for each database and applied using appropriate filters related to publication type, language and subject area. The complete electronic search strategy for each database, including Boolean operators and filters, is provided in Appendix A to enhance transparency and reproducibility.

The literature search was conducted through January 2026, and searches were limited to studies published in English or Turkish.

Studies were included if they were peer‐reviewed quantitative research articles published in English or Turkish; involved participants diagnosed with both an eating disorder and borderline personality disorder; examined the effects of dialectical behaviour therapy or DBT‐based interventions; and reported quantitative outcomes related to eating disorder symptoms, borderline personality disorder symptoms, or both.

Studies were excluded if they were review articles, meta‐analyses or theoretical papers; examined only borderline personality disorder or only eating disorders without comorbidity; did not evaluate DBT as the primary intervention; were non‐research documents; or lacked sufficient methodological detail.

Risk of bias was assessed using the Joanna Briggs Institute (JBI, Aromataris and Munn 2020) critical appraisal checklists appropriate for each study design (e.g., case series and quasi‐experimental studies). Two reviewers independently conducted the quality appraisal, and disagreements were resolved through discussion until consensus was reached. The methodological quality of the included studies is presented in Table 1.

**TABLE 1:** Methodological quality assessment of studies.

Authors	Study type	Clear inclusion criteria?	Valid identification of condition?	Reliable outcome measurement?	Clear reporting of outcomes?	Appropriate statistical analysis?	Adequate follow‐up?	Overall risk rating
Chen et al. (2008)	Quasi‐experimental/pre–post	Yes	Yes	Yes	Yes	No	Yes	Low
Palmer et al. (2003)	Quasi‐experimental/pre–post	No	Yes	No	Yes	No	Unclear	High
Kröger et al. (2010)	Quasi‐experimental/pre–post	Yes	Yes	Yes	Yes	Yes	Yes	Low
Navarro‐Haro et al. (2018)	Quasi‐experimental/pre–post	Yes	Yes	Yes	Yes	Yes	No	Low‐moderate
Navarro‐Haro et al. (2021)	Quasi‐experimental/pre–post	Yes	Yes	Yes	Yes	Yes	Yes	Low
Navarro‐Haro et al. (2025)	Single case	Yes	Yes	Yes	Yes	Yes	No	Low‐moderate
Denning et al. (2024)	Case series/longitudinal	Yes	Yes	Yes	Yes	Yes	Yes	Low
Liakopoulou et al. (2023)	Quasi‐experimental/pre–post	Yes	Yes	Yes	Yes	Yes	No	Moderate

*Note:* Study type: research design of the study. Clear inclusion criteria: explicit participant selection and diagnostic criteria. Valid identification of condition: use of standardized diagnostic or clinical assessment procedures. Reliable outcome measurement: use of validated instruments. Clear reporting of outcomes: sufficient description of pre‐ and post‐treatment results. Appropriate statistical analysis: suitable statistical methods for the study design. Adequate follow‐up: at least one assessment beyond posttreatment. Overall risk rating: based on the number of unmet methodological criteria. For each item, two independent reviewers assessed the studies and marked them as Yes/No/Unclear/NA and rated the overall risk between Low to High.

Owing to substantial heterogeneity in study design, diagnostic composition, outcome measures and reporting formats, a quantitative meta‐analysis was not considered appropriate. Therefore, a structured narrative synthesis was conducted.

The study selection process began with the identification of records through database searches. A total of 6053 records were identified, including 5 from Wiley, 716 from Web of Science, 4289 from SpringerLink and 1043 from Scopus. After the removal of 16 duplicate records through manual comparison of titles, authors and publication details, 6037 unique records remained for title and abstract screening. No records were identified through registers.

**FIGURE 1:**
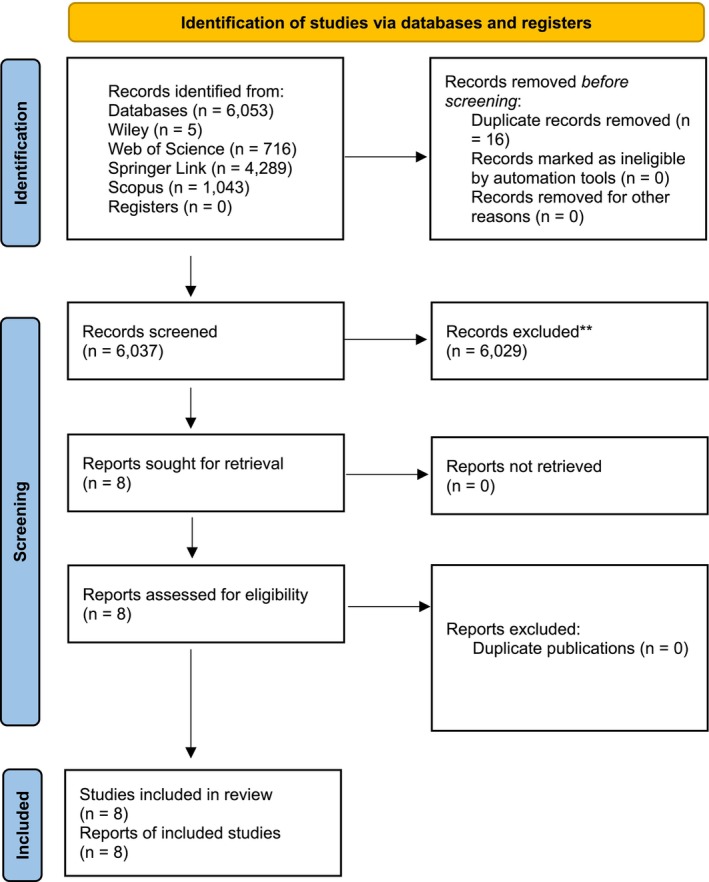
PRISMA flow diagram.

Following title and abstract screening, 6029 records were excluded because they did not meet the predefined eligibility criteria. The full texts of the remaining eight reports were retrieved and assessed for eligibility. No reports were excluded because of retrieval issues. All eight studies met the inclusion criteria and were included in the final systematic review. Titles, abstracts and full‐text articles were independently screened by two reviewers. Disagreements were resolved through discussion until consensus was reached, and inter‐rater agreement exceeded 90%. The study selection process is illustrated in Figure 1, and the methodological quality assessment is presented in Table 1.

## 3 Results

This systematic review included eight studies that investigated the effects of dialectical behaviour therapy (DBT) on eating disorder (ED) outcomes among individuals with comorbid borderline personality disorder (BPD). The included studies showed substantial heterogeneity in study design, sample size, treatment duration, outcome measures and analytical methods. The study designs encompassed single‐case reports, uncontrolled pre–post observational studies, naturalistic longitudinal investigations and non‐randomized comparative studies. The most common outcomes assessed were eating disorder symptom severity, maladaptive eating behaviours (including binge eating, purging, and restriction), self‐harm and suicidality, emotion regulation and general psychosocial functioning. A limited number of studies have examined predictor, moderator and interaction effects related to baseline BPD symptom severity. The study‐specific findings are presented below, followed by a comparative synthesis. The characteristics, outcomes and risk of bias of the included studies are presented in Table 2. An overview of the characteristics and key outcomes of the included studies is presented in Table 3.

**TABLE 2:** Characteristics and outcomes of included studies.

Authors	Sample	Age *M* (SD)	BPD measures	ED measures	Key results
Chen et al. (2008)	*N* = 8 women; BED/BN; 6‐month DBT; USA	Median = 31	SCID‐II; self‐report	EDE	Large reductions in binge eating and ED severity; approximately 50% binge abstinence at post and follow‐up
Palmer et al. (2003)	*N* = 7 women; treatment‐resistant ED + BPD; UK	NR	Clinical records	Clinical records	Reduced hospital days and severe self‐harm; most no longer met ED criteria at follow‐up
Kröger et al. (2010)	*N* = 24 women; AN/BN; inpatient DBT + CBT‐ED; Germany	31.1 (7.6)	SCID‐II (criteria count)	EDI‐2; BMI	Reduced binge eating (BN); BMI increase at follow‐up (AN); remission: BN 54%, AN 33%
Navarro‐Haro et al. (2018)	*N* = 118 women; DBT vs. TAU‐CBT; Spain	27.37 (8.75)	Clinical/self‐report	ED behaviours	Both treatments reduced ED behaviours; DBT greater improvement in BPD‐related dysfunction
Navarro‐Haro et al. (2021)	*N* = 109 women; DBT vs. TAU‐CBT; Spain	27.38 (NR)	BDI‐II; ERQ; BIS‐11	EES	Emotional eating decreased over time; no treatment × time effects; gains maintained long‐term
Denning et al. (2024)	*N* = 176 adults; DBT‐based partial hospitalization; USA	25.9 (9.1)	BEST	EDE‐Q	Higher baseline BPD predicted greater ED severity but faster early reductions; no BPD × time effects
Liakopoulou et al. (2023)	*N* = 72; AN/BN; 12‐month DBT; Greece	26.7 (6.7)	BSL‐23	EDE‐Q	Significant reductions in ED severity, BPD symptoms, and suicidal ideation
Navarro‐Haro et al. (2025)	Single female case; EDNOS; 12‐month DBT; Spain	31	SCID‐II	Clinical/self‐report	No longer met BPD criteria; reduced impulsivity and ED symptoms

Abbreviations: AN = anorexia nervosa; BED = binge‐eating disorder; BN = bulimia nervosa; BPD = borderline personality disorder; DBT = dialectical behaviour therapy; ED = eating disorder; EDNOS = eating disorder not otherwise specified; *M* = mean; NR = not reported; NSSI = non‐suicidal self‐injury; SD = standard deviation; TAU‐CBT = treatment‐as‐usual cognitive behavioural therapy.

**TABLE 3:** Summary of studies examining the effects of DBT on eating disorder outcomes in individuals with comorbid BPD.

Study	Sample (*n*)	ED type	DBT duration	Design	Treatment format and concurrent interventions	Key findings
Palmer et al. (2003)	7	Mixed	Not specified (variable)	Naturalistic observational	DBT programme; additional treatment components not clearly reported	Reductions in hospital days, eating disorder severity, and self‐harm behaviours
Chen et al. (2008)	8	BED/BN	Standard DBT (6 months)	Case series	Standard comprehensive DBT; additional interventions not reported	Reductions in binge eating and purging; improvements in social functioning
Kröger et al. (2010)	24	AN/BN	3 months (inpatient) + CBT module	Observational	Inpatient DBT combined with a CBT‐based eating‐disorder module	Reduced binge eating in BN; partial increases in BMI in AN; improved psychosocial functioning
Navarro‐Haro et al. (2018)	118	Mixed	6 months	Non‐randomized comparison	DBT programme compared with TAU‐CBT; additional concurrent interventions not clearly reported	Reductions in binge eating, purging, and restrictive behaviours
Navarro‐Haro et al. (2021)	109	Mixed	6 months	Non‐randomized longitudinal	DBT programme compared with TAU‐CBT; additional concurrent interventions not clearly reported	Sustained reductions in emotional eating in the DBT condition
Liakopoulou et al. (2023)	72	AN/BN	12 months	Pre‐post	DBT programme; concurrent interventions not clearly reported	Reductions in eating disorder severity, BPD symptoms, and suicidal ideation
Denning et al. (2024)	176	Mixed	Partial hospitalization (variable)	Longitudinal	DBT‐based partial hospitalization programme; treatment was delivered within a higher‐level multidisciplinary care setting	Decreases in overall ED severity, binge eating, fasting, and self‐harm behaviours
Navarro‐Haro et al. (2025)	1	EDNOS	12 months	Single‐case	Individual DBT case treatment; additional interventions not clearly reported	Reductions in binge eating and impulsivity; improvements in emotion regulation

*Note:* ‘Not clearly reported’ indicates that the original study did not provide sufficient information to determine whether additional interventions were delivered alongside DBT. Reported changes reflect differences from baseline to post‐treatment or follow‐up as described in the original studies.

Abbreviations: AN = anorexia nervosa; BED = binge‐eating disorder; BN = bulimia nervosa; DBT = dialectical behaviour therapy; ED = eating disorder; EDNOS = eating disorder not otherwise specified; TAU‐CBT = treatment‐as‐usual cognitive behavioural therapy.

Palmer et al. (2003) investigated the efficacy of DBT in seven female patients with eating disorders and comorbid BPD who were described as treatment resistant. The study employed a naturalistic observational design without a control group, using clinical records rather than standardized psychometric instruments. Outcomes were assessed across three periods: the 18 months before DBT, during treatment and up to 18 months following treatment. Due to the limited sample size and variability in available data, no formal statistical analyses were conducted. During the follow‐up period, reductions were observed in the number of hospital days, the severity of eating disorder presentations and both severe and mild self‐harming behaviours requiring medical attention. The relationship between baseline BPD symptom severity and treatment outcomes was not quantitatively examined.

Chen et al. (2008) examined the effects of standard DBT in a descriptive clinical case series involving women with comorbid BPD and binge‐eating disorder (BED; *n* = 5) or bulimia nervosa (BN; *n* = 3). Results indicated substantial reductions in objective binge eating frequency and overall eating disorder severity from pre‐ to post‐treatment, with large effect sizes (*d* = 1.07 for binge eating; *d* = 1.66 for total EDE score). At the 6‐month follow‐up, these improvements were sustained or further strengthened (*d* = 2.00 and *d* = 0.97, respectively). Among participants with BN, marked reductions in purging behaviours were observed, with most individuals achieving abstinence by post‐treatment or follow‐up. Improvements in social functioning and reductions in non–eating disorder Axis I comorbidity were reported. Decreases in suicidal and non‐suicidal self‐injurious behaviours were also observed, particularly at follow‐up. Changes in BMI were variable and did not demonstrate a consistent pattern across participants. The study did not examine predictor or moderator effects, including the role of BPD symptom severity. Treatment adherence, attrition and medication use were not reported as study outcomes.

Kröger et al. (2010) examined the outcomes of an adapted inpatient dialectical behaviour therapy (DBT) programme with an additional cognitive–behavioural module for eating disorders in a clinical sample of women diagnosed with borderline personality disorder (BPD) and comorbid anorexia nervosa (AN) or bulimia nervosa (BN) who had previously failed to respond to eating disorder specific treatments (*N* = 24). Following a 3‐month inpatient DBT intervention, outcomes were evaluated at pretreatment, post‐treatment and 15‐month follow‐up. With respect to eating disorder outcomes, women diagnosed with BN exhibited a significant decrease in binge‐eating episodes following treatment, which persisted at follow‐up (*p* < 0.001), together with moderate to large improvements in eating‐disorder–related symptoms over time. In contrast, for women with AN, body mass index did not increase significantly at post‐treatment, although a significant improvement emerged by follow up (*p* = 0.032), accompanied by large effect sizes for reductions in eating‐disorder symptom severity. Remission rates at follow up were higher for BN (53.8%) than for AN (38%), with a substantial proportion of participants with AN crossing over to BN during follow‐up. Significant improvements in general psychopathology and global psychosocial functioning were observed at post‐treatment among individuals with BN and were maintained over follow‐up, while participants with AN exhibited significant change mainly during the follow‐up phase. No significant reductions were observed in BPD symptom severity for either subgroup, although reductions in mood disorder comorbidity were reported at follow‐up.

In a naturalistic clinical setting, Navarro‐Haro et al. (2018) assessed the effectiveness of standard DBT compared with treatment‐as‐usual cognitive behavioural therapy (TAU‐CBT). The study employed a non‐randomized two‐group pre–post design involving 118 female patients (DBT = 71; TAU‐CBT = 47) receiving day hospital or outpatient treatment. Assessments were conducted at pretreatment and post‐treatment following a 6‐month intervention period. Both treatment groups demonstrated decreases in dysfunctional eating behaviours, including binge eating, purging and restriction. However, the time × treatment interaction was not statistically significant, indicating comparable reductions across DBT and TAU‐CBT. The study did not assess whether initial levels of BPD symptom severity were associated with eating disorder outcomes. Secondary outcomes, including treatment adherence, remission and DBT skills use, were not analysed, and dropout rates were reported descriptively without statistical comparison.

Navarro‐Haro et al. (2021) examined the long‐term effectiveness of standard dialectical behaviour therapy (DBT) compared with treatment‐as‐usual cognitive behavioural therapy (TAU‐CBT) in a clinical sample of women diagnosed with comorbid borderline personality disorder and eating disorders (AN, BN, EDNOS; *N* = 109). Participants received a 6‐month outpatient or day‐hospital intervention (DBT = 64; TAU‐CBT = 45), with assessments conducted at pretreatment, post‐treatment and at 4‐ and 6‐year follow‐ups. Regarding outcomes related to eating disorders, emotional eating showed a significant reduction across the DBT condition from baseline through long‐term follow‐up assessments. However, no significant differences were observed between groups in the change over time in emotional eating. With respect to secondary outcomes associated with emotion dysregulation, individuals receiving DBT exhibited sustained improvements in depression, trait anger, impulsivity, resilience and expressive suppression, whereas improvements in the TAU‐CBT group were largely confined to depression, resilience and trait anger. Mixed‐effects analyses revealed no significant treatment × time interactions for any outcome, indicating comparable symptom trajectories across DBT and TAU‐CBT conditions over the 6‐year follow‐up period. However, reliable change analyses showed that a higher proportion of participants in the DBT condition achieved clinically meaningful improvement over time, particularly for depression, resilience, and emotion regulation indices. Overall, the findings suggest that standard DBT is associated with sustained long‐term improvements in emotion‐related and eating‐related outcomes in individuals with comorbid BPD and eating disorders, although its long‐term effectiveness did not significantly differ from that of TAU‐CBT in this naturalistic setting.

Liakopoulou et al. (2023) conducted a single‐group uncontrolled pre‐post study in which 72 patients with comorbid borderline personality disorder (BPD) and eating disorders participated in a 12‐month standard dialectical behaviour therapy (DBT) programme at a university hospital. The sample of this study consisted primarily of female patients (90.3%), with diagnoses of anorexia nervosa (16.7%) and bulimia nervosa (83.3%), while patients with binge‐eating disorder were excluded from the study. The BSL‐23, EDE‐Q, SBQ and DBT‐WCCL were used at baseline and post‐treatment. Significant reductions in eating disorder symptom severity, BPD symptoms and suicidal ideation were found, as indicated by paired‐samples *t*‐tests and Wilcoxon signed‐rank tests (*p* < 0.001). At both baseline and post‐treatment, a positive correlation was identified between ED symptom severity and BPD symptom severity. No analyses were conducted to examine BPD × time interactions or moderation effects.

Denning et al. (2024) examined whether baseline BPD symptom severity influenced ED treatment outcomes in a naturalistic longitudinal sample of 176 adults receiving DBT‐based partial hospitalization treatment. Assessments were conducted at admission, 1 month, discharge and 6‐month follow‐up. Overall, ED symptom severity decreased over time, while higher baseline BPD symptoms predicted greater ED severity across treatment. Change in overall ED severity did not differ as a function of BPD × time. In contrast, binge eating and fasting behaviours showed steeper early reductions during the first month of treatment among individuals with higher BPD symptom severity. Baseline BPD symptoms also predicted higher parasuicidal behaviour, with significant BPD × time interactions indicating greater reductions in self‐harm behaviours among those with elevated BPD symptoms. No significant effects were observed for DBT skills use, remission, relapse or treatment retention.

Navarro‐Haro et al. (2025) demonstrated a single‐case study involving a 31‐year‐old female patient with comorbid EDNOS and BPD who completed a 12‐month outpatient standard DBT programme. A single‐case uncontrolled pre–post design was employed, with assessments conducted using semi‐structured interviews and self‐report measures at pre‐ and post‐treatment. Clinically meaningful reductions were observed in transdiagnostic symptoms associated with BPD and EDNOS, including emotion regulation difficulties, impulsivity and binge‐eating behaviours. No formal statistical analyses, effect sizes or moderator effects were reported.

Overall, the included studies consistently suggest that DBT‐based interventions are associated with reductions in eating disorder symptom severity, maladaptive eating behaviours and self‐harm. Although baseline BPD symptom severity was examined in a limited number of studies, it did not consistently predict treatment outcomes.

## 4 Discussion

This systematic review synthesized findings from eight studies examining DBT and DBT‐based interventions for individuals with comorbid borderline personality disorder (BPD) and eating disorders (EDs). Overall, the included studies reported improvements in eating disorder symptom severity, maladaptive eating behaviours and, where assessed, self‐harm–related outcomes. However, the strength of these findings is limited by substantial heterogeneity in study design, diagnostic composition, treatment delivery and outcome measurement. Accordingly, the current evidence suggests potential clinical utility of DBT‐based interventions in this population, while not allowing for firm causal conclusions regarding treatment efficacy.

Across the limited number of studies examining baseline BPD symptom severity, findings did not consistently indicate poorer eating disorder outcomes among individuals with higher symptom levels. In some studies, greater baseline BPD severity was associated with steeper early reductions in binge eating and self‐harming behaviours. One possible explanation is that individuals with more severe emotion dysregulation and impulsivity may have greater room for observable clinical improvement and may benefit more directly from DBT modules targeting these processes. However, this interpretation remains tentative, as regression to the mean, variability in treatment intensity and baseline clinical severity may also account for these patterns. Further research is required to clarify whether BPD severity functions as a reliable moderator of treatment response.

Interpretation of findings should also consider treatment specificity. Several included studies delivered DBT within integrated or higher levels of care, often combined with additional interventions such as cognitive behavioural therapy for eating disorders, nutritional rehabilitation, pharmacotherapy or multidisciplinary inpatient programmes. As a result, observed improvements cannot be attributed exclusively to DBT components. Rather, the evidence suggests that DBT‐informed interventions may be most effective when embedded within comprehensive treatment frameworks for complex clinical presentations involving both BPD and EDs. Future research should more precisely delineate the unique contribution of DBT components by controlling for concurrent interventions.

Although not eligible for inclusion in the present review because participants were not uniformly diagnosed with confirmed comorbid BPD and eating disorders, previous work by Federici et al. (2012) and Federici and Wisniewski (2013) provides valuable context for interpreting the current findings. Federici et al. (2012) described the development of an intensive DBT‐informed programme for individuals with complex, multidiagnostic eating disorder presentations, emphasizing the integration of standard DBT with evidence‐based eating disorder interventions, including nutritional rehabilitation, meal support, exposure‐based strategies and cognitive‐behavioural techniques. Building on this treatment model, Federici and Wisniewski (2013) subsequently reported clinically meaningful improvements in eating disorder symptoms, self‐injurious behaviours and overall functioning in a case series of individuals receiving the programme. Collectively, these studies further support the potential value of integrated DBT‐informed approaches for individuals with severe and treatment‐resistant eating disorders, while also reinforcing that favourable outcomes in complex clinical populations are likely to reflect the combined effects of multidisciplinary treatment rather than DBT components alone. This distinction highlights the importance of differentiating studies involving heterogeneous eating disorder populations from those specifically examining individuals with confirmed BPD–ED comorbidity.

The clinical applicability of standard DBT may also vary according to emotion regulation profiles across eating disorder presentations. Standard DBT was originally developed to address pervasive emotion dysregulation, behavioural impulsivity and self‐harm and may therefore be particularly relevant for undercontrolled presentations more commonly observed in some individuals with bulimia nervosa and binge eating disorder. In these cases, maladaptive eating behaviours may function as strategies to regulate intense negative affect or interpersonal distress. However, substantial heterogeneity exists both within and across diagnostic categories, and emotion dysregulation processes cannot be fully captured by DSM classification alone (Lavender et al. 2015).

In contrast, restrictive anorexia nervosa presentations are more frequently associated with behavioural rigidity, perfectionism, emotional inhibition and overcontrolled coping styles. These features may reduce the fit of standard DBT when delivered as a stand‐alone intervention, particularly in individuals whose psychopathology is primarily characterized by emotional constriction rather than impulsive dysregulation. This interpretation should be considered cautiously given the limited empirical evidence directly comparing treatment response across eating disorder subtypes (Rozakou‐Soumalia et al. 2021).

Radically open dialectical behaviour therapy (RO‐DBT) offers a complementary framework for understanding and targeting overcontrolled emotional and behavioural patterns. RO‐DBT specifically focuses on maladaptive overcontrol, including emotional inhibition, cognitive rigidity and social withdrawal (Lynch et al. 2015). Although RO‐DBT was beyond the scope of the present review, integrating insights from both DBT and RO‐DBT may enhance transdiagnostic models of eating disorders with comorbid personality pathology. Future research should examine whether emotion regulation style, rather than diagnostic category alone, moderates response to DBT, DBT‐informed interventions and RO‐DBT.

### 4.1 Limitations and Future Directions

Several limitations should be considered when interpreting the findings of this systematic review. All included studies employed uncontrolled or non‐randomized designs, limiting causal inferences regarding the effectiveness of DBT in comorbid BPD–ED populations. In addition, considerable heterogeneity in treatment modalities, outcome measures and follow‐up periods reduced cross‐study comparability and precluded quantitative synthesis.

Importantly, DBT was not consistently delivered as a stand‐alone intervention. In several studies, DBT was implemented within integrated or higher level care settings and combined with additional treatment components, making it difficult to isolate its specific effects on eating disorder outcomes. This methodological limitation significantly constrains interpretation of treatment efficacy.

Few studies systematically examined moderators or mechanisms of change. Although some investigated baseline BPD symptom severity as a predictor, findings were limited and inconsistent. Potential mechanisms such as emotion regulation, impulsivity and DBT skills acquisition were rarely assessed, leaving the processes underlying treatment effects insufficiently understood.

Future research should prioritize well‐powered randomized controlled trials specifically targeting comorbid BPD and ED populations. Standardized outcome measures, consistent follow‐up intervals and systematic evaluation of mediators and moderators would improve comparability across studies. In addition, comparative designs examining DBT alongside other integrated or transdiagnostic interventions, as well as studies isolating DBT components from concurrent treatments, are needed to clarify mechanisms of change and optimize clinical decision‐making. Studies directly comparing standard DBT, integrated DBT programmes and other transdiagnostic approaches would be particularly valuable for clarifying the active therapeutic ingredients underlying treatment response.

### 4.2 Clinical Implications

The findings of the present review suggest that DBT‐informed interventions may be clinically useful for individuals with comorbid BPD and EDs, particularly when emotion dysregulation, impulsivity, self‐harm or affect‐driven eating behaviours are prominent. Clinicians working with high‐risk comorbid populations may benefit from treatment approaches that prioritize life‐threatening and therapy‐interfering behaviours while also addressing ED symptoms. DBT skills training may be particularly relevant for supporting emotion regulation and behavioural stabilization within comprehensive care settings. However, treatment planning should consider the heterogeneity of ED presentations and emotion regulation profiles. Individuals with more pronounced undercontrolled patterns may be particularly well aligned with standard DBT, whereas those characterized by rigidity, emotional inhibition, or overcontrol may require adapted or complementary interventions.

## 5 Conclusion

The available literature suggests that DBT and DBT‐based interventions may be associated with improvements in eating disorder symptoms and high‐risk behaviours among individuals with comorbid BPD and EDs. However, the evidence base remains limited by uncontrolled and non‐randomized study designs, heterogeneous treatment formats and the frequent use of integrated interventions. Accordingly, improvements in ED outcomes cannot be attributed exclusively to DBT components. Future research using rigorous controlled designs is needed to clarify the effectiveness of DBT, identify mechanisms of change and determine whether treatment response differs according to ED presentation and emotion regulation profile. Despite these limitations, DBT‐informed approaches may offer a clinically relevant framework within comprehensive care for individuals with complex comorbid BPD–ED presentations.

## Author Contributions

All authors contributed equally to the conceptualization, methodology, investigation, writing of the original draft and review and editing of the manuscript. All authors read and approved the final version of the manuscript.

## Funding

The authors have nothing to report.

## Disclosure

This review was not preregistered.

## Conflicts of Interest

The authors declare no conflicts of interest.

## Data Availability

Data sharing is not applicable to this article as no datasets were generated or analysed during the current study.

## Appendix A Full Search Strategy String

(“dialectical behavior therapy” OR DBT) AND

(“borderline personality disorder” OR BPD) AND

(“eating disorder” OR “bulimia nervosa” OR “binge eating disorder” OR “anorexia nervosa”)
